# Large Epiphrenic Esophageal Diverticulum: An Unexpected Trap at the Distal Esophagus

**DOI:** 10.7759/cureus.107348

**Published:** 2026-04-19

**Authors:** Rebecca E Aquino, Kshitij Thakur, Aaron Brenner

**Affiliations:** 1 Internal Medicine, University of Kentucky, Lexington, USA; 2 Gastroenterology and Hepatology, University of Kentucky, Lexington, USA

**Keywords:** epiphrenic esophageal diverticulum, esophageal dysmotility, heller myotomy, peroral endoscopic myotomy (poem), pulsion diverticulum

## Abstract

Esophageal diverticula are rare and typically asymptomatic, but when symptomatic, they can present with life-threatening complications. They are categorized based on location and esophageal layer involvement. Their underlying pathophysiology involves either a traction or a pulsion mechanism. Epiphrenic diverticula lack a muscular layer and are typically caused by pulsion secondary to an underlying motility disorder. The underlying anatomy and pathophysiology help determine the most appropriate management approach. Treatment includes a range of endoscopic and surgical techniques and must be individualized on a case-by-case basis.

## Introduction

Esophageal diverticula are rare outpouchings of the esophageal mucosa, occurring in approximately 0.06-4 percent of the population [1]. They are often asymptomatic and do not require treatment; however, complications can be life-threatening and include esophageal obstruction, perforation, and squamous cell carcinoma [2]. Here, we present a case of a large epiphrenic esophageal diverticulum complicated by esophageal obstruction requiring urgent upper endoscopy.

## Case presentation

**Figure 1:**
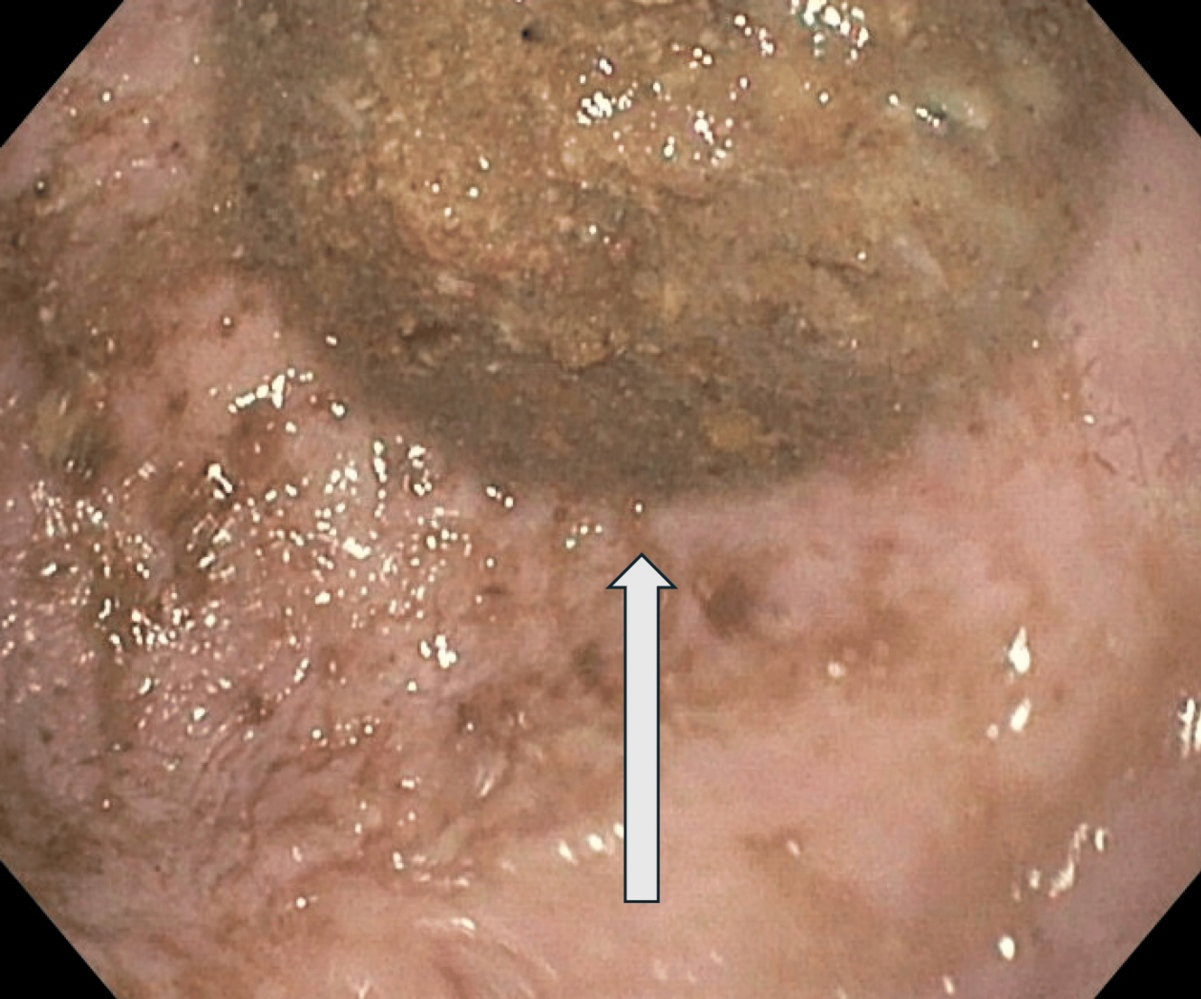
Single large diverticulum full of solid and liquid debris (white arrow).

A 77-year-old man with a history of atrial fibrillation, chronic obstructive pulmonary disease, gastroesophageal reflux disease, and chronic dysphagia for two years presented with progressively worsening dysphagia. The patient had been unable to tolerate solid foods for one week and was having frequent regurgitation with attempted intake by mouth. Computed tomography (CT) scan showed evidence of esophageal obstruction with food material, dilation of the mid to distal esophagus up to 6.9 × 5 cm, distal esophageal decompression, and backup of fluid to the level of the cervicothoracic junction. The patient was taken for urgent esophagogastroduodenoscopy (EGD) on the same day, which revealed a single large diverticulum 6 cm deep and full of solid and liquid food material (Figure 1).

The food material was removed with suctioning. The diverticulum was noted to be causing 8 cm of severe luminal narrowing in the distal esophagus. This stricture was not traversable with a regular gastroscope and required exchange with an ultra-slim gastroscope, which was used to measure the area of narrowing from 40 cm to 48 cm from the incisor.

**Figure 2:**
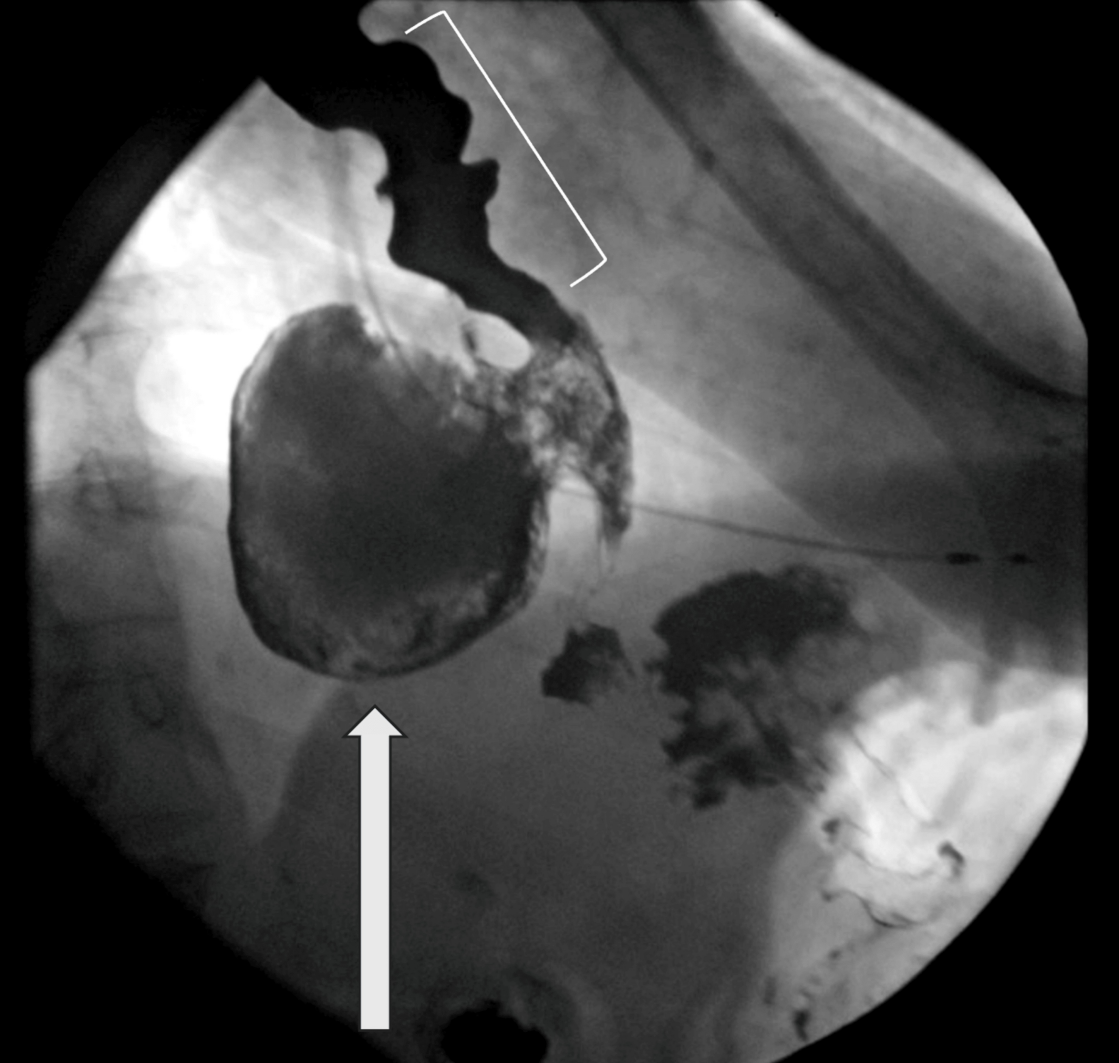
Barium swallow showing esophageal dysmotility with tertiary contractions (white bracket) and a large distal esophageal diverticulum (white arrow). Proximal structures correspond with the top left corner of the figure, while distal structures correspond with the bottom right corner.

After the procedure, the patient was able to tolerate a liquid diet. After discharge from the hospital, the patient completed a barium swallow, which demonstrated esophageal dysmotility with tertiary contractions (Figure 2).

The diverticulum was initially presumed to be a hernia; however, comparison with CT imaging confirmed the diagnosis of esophageal diverticulum. The presence of esophageal dysmotility indicated that this was likely a pulsion diverticulum.

**Figure 3:**
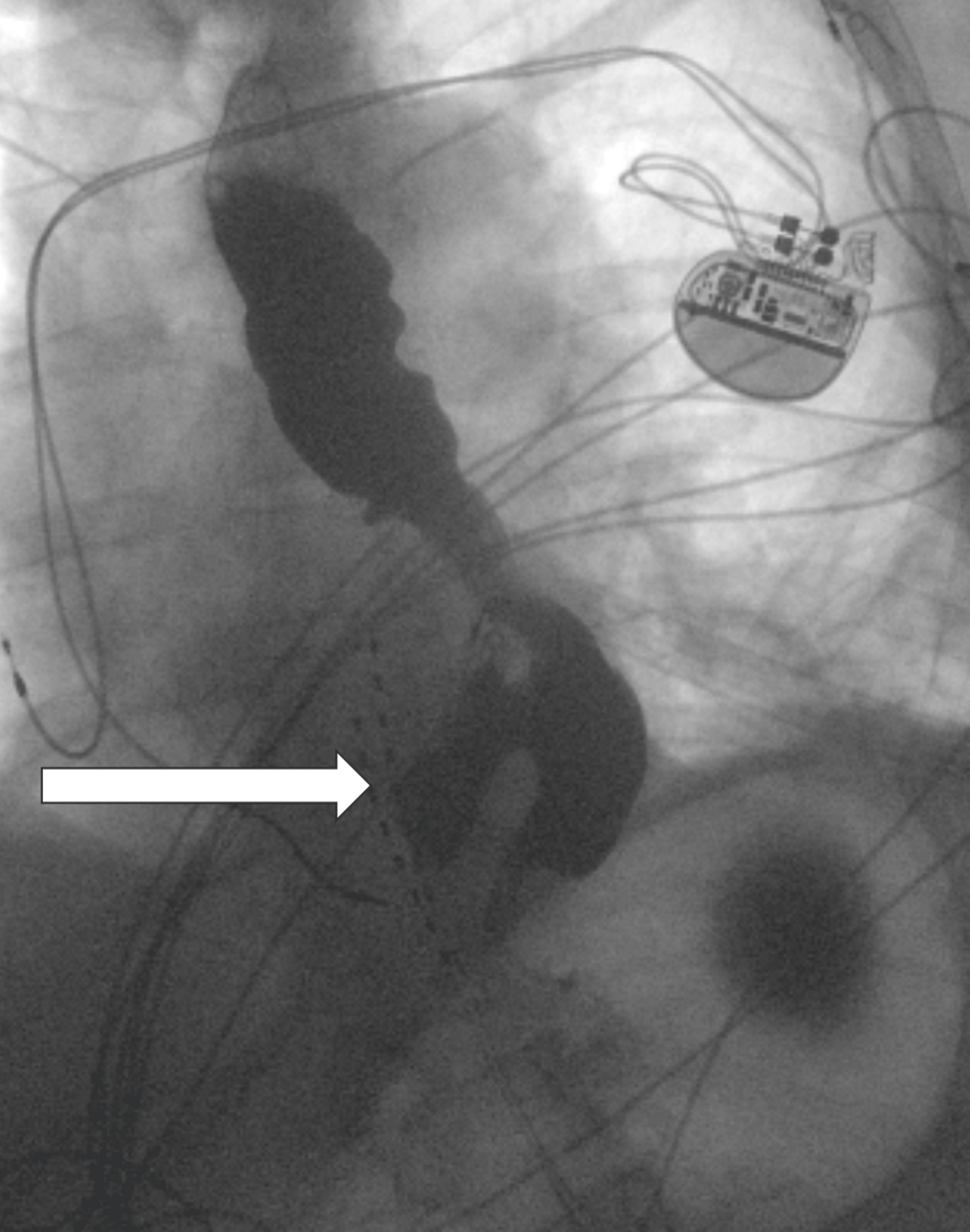
Post-operative barium swallow showing residual, but much smaller, epiphrenic esophageal diverticulum.

The patient was referred to thoracic surgery for definitive management. Diverticular peroral endoscopic myotomy (D-POEM) was considered; however, it was felt that the outward pouch of the diverticulum was too large, and this procedure would result in a large esophageal lumen with a high risk of perforation. The patient subsequently underwent left thoracotomy, resection of the esophageal diverticulum, and Heller myotomy. The myotomy was performed proximally 180 degrees from the diverticulectomy and extended from the sixth intercostal space to the gastric wall. Post-operative barium swallow showed residual, but much smaller, epiphrenic esophageal diverticulum (Figure 3).

On follow-up, the patient reported significant improvement in symptoms.

## Discussion

Esophageal diverticula typically occur in older adults but are overall rare. Radiology studies show an incidence rate of up to four percent, but the majority of those are asymptomatic [1]. They are classified based on location within the esophagus and esophageal layer involvement. Zenker's diverticulum, or pharyngeal diverticulum, is the most common type, occurring in the proximal esophagus and containing mucosa and submucosa. Similarly, epiphrenic diverticula usually contain mucosa and submucosa but are located within 10 cm of the gastroesophageal junction. Due to their usual absence of muscularis propria, pharyngeal and epiphrenic diverticula are called false diverticula [2]. Epiphrenic diverticula are exceedingly rare, making up less than 10 percent of all esophageal diverticula [1].

The patients with esophageal diverticula are typically asymptomatic. When symptoms are present, they may include dysphagia, regurgitation, and aspiration [2]. As the diverticula grow larger, additional symptoms may be caused by the compression of surrounding structures [1]. The diagnosis is made using barium swallow combined with continuous fluoroscopy. This method allows for the localization of the esophageal outpouching as it is filled with barium contrast. Barium swallow is also useful for defining the patient's anatomy, which could be useful for surgical or endoscopic planning [2].

Esophageal diverticula develop via two basic mechanisms: traction and pulsion. Traction diverticula form as a result of external force on the esophagus [2]. Pulsion diverticula are more common in patients with esophageal motility disorders owing to the presence of increased intraluminal pressure and the resulting herniation of the esophagus [3]. Underlying motility disorders are important to diagnose prior to the definitive management of pulsion diverticula, as they may lead to recurrent symptoms [4]. It is unclear why manometry was not included in this patient's workup, but it is generally recommended prior to the decision of treatment approach [4].

Treatment is warranted in symptomatic patients with esophageal diverticula and even in some asymptomatic patients. Surgical treatment options most often include diverticulectomy, myotomy, and fundoplication, which can be approached by thoracotomy or laparoscopically [3]. Endoscopic management is done by peroral endoscopic myotomy, which is becoming more popular as it is effective, safe, and less invasive than surgical approaches [4]. Determining the best approach requires the careful consideration of the endoscopic view of the diverticulum, the patient's body habitus, the positioning of the diverticulum, local expertise, and the size of the diverticulum [2].

## Conclusions

Ultimately, the best approach for the individual patient depends on their anatomy, comorbidities, and characteristics of the diverticulum itself. Comprehensive workup prior to surgical or endoscopic management is key to the success of the intervention. Further, the proper assessment of the patient's symptom burden is possibly the most important step. Treatment should be individualized on a case-by-case basis.
